# Screening, Expression, and Identification of Nanobody against SARS-CoV-2 Spike Protein

**DOI:** 10.3390/cells11213355

**Published:** 2022-10-24

**Authors:** Qianling Su, Wei Shi, Xianing Huang, Yakun Wan, Guanghui Li, Bengang Xing, Zhi Ping Xu, Hongbo Liu, Bruce D. Hammock, Xiaomei Yang, Shihua Yin, Xiaoling Lu

**Affiliations:** 1 College of Stomatology/Hospital of Stomatology/Guangxi Key Laboratory of Nanobody Research/Guangxi Nanobody Engineering Research Center/Laboratory Animal Center/School of Basic Medical Sciences/The Second Clinical Medical College, Guangxi Medical University, Nanning 530021, China; 2 Shanghai Novamab Biopharmaceuticals Co., Ltd., Shanghai 201203, China; 3 Division of Chemistry and Biological Chemistry, School of Physical and Mathematical Sciences, Nanyang Technological University, Singapore 637551, Singapore; 4 Australian Institute for Bioengineering and Nanotechnology, University of Queensland, Brisbane, QLD 4072, Australia; 5 Department of Laboratory Medicine, The Second Affiliated Hospital of Guilin Medical University, Guilin 541000, China; 6 UCD Comprehensive Cancer Center, Department of Entomology and Nematology, University of California, Davis, CA 95616, USA

**Keywords:** SARS-CoV-2, spike protein, nanobody, single-domain antibody, phage display

## Abstract

Coronavirus disease 2019 (COVID-19) is caused by the severe acute respiratory syndrome coronavirus 2 (SARS-CoV-2), an infectious disease that has become a serious burden on global public health. This study screened and yielded specific nanobodies (Nbs) against SARS-CoV-2 spike protein receptor binding domain (RBD), following testing its basic characteristics. A nanobody phage library was established by immunizing a camel with RBD protein. After three rounds of panning, the positive colonies were screened by enzyme-linked immunosorbent assay (ELISA). By sequencing, four different sequences of nanobody gene fragments were selected. The four nanobody fusion proteins were expressed and purified, respectively. The specificity and affinity of the four nanobodies were identified by ELISA. Our results showed that an immune phage display library against SARS-CoV-2 has been successfully constructed with a library capacity of which was 4.7 × 10^8^ CFU. The four purified nanobodies showed specific high-affinity binding SARS-CoV-2 S-RBD. Among these, the antigen binding affinity of Nb61 was more comparable to that of commercial rabbit anti-SARS-CoV-2 S-RBD antibodies. In sum, our study has obtained four nanobody strains against SARS-CoV-2 S-RBD with significant affinity and specificity, therefore laying an essential foundation for further research as well as the applications of diagnostic and therapeutic tools of SARS-CoV-2.

## 1. Introduction

Severe acute respiratory syndrome coronavirus 2 (SARS-CoV-2) is the etiologic agent of Coronavirus disease 2019 (COVID-19) [1]. Clinical manifestations of COVID-19 in the general population range from asymptomatic infection, fever, cough, hemoptysis, respiratory failure, and even death [2]. As of 4 July 2022, this highly contagious virus has caused more than 545 million infections and claimed over 6.3 million lives (https://covid19.who.int, accessed on 4 July 2022). The continued spread and mutations of SARS-CoV-2 have had a profound impact on global public health, economic activity, and social order, posing a huge challenge to national healthcare systems. Therefore, additional diagnostic tools and effective therapeutics remain urgently needed.

SARS-CoV-2 is a single-strand positive-sense RNA virus belonging to the family Coronaviridae and Betacoronavirus genus [3,4]. As the critical function for binding to the human angiotensin-converting enzyme 2 (hACE2) and cell entry, the receptor binding domain (RBD) of the spike (S) protein in SARS-CoV-2 plays an important role in mediating virus adsorption and infection of host cells. Therefore, the S protein is the most preferred antigen target for the research and development of detection kits, vaccines, neutralizing antibodies, and virus inhibitors [5]. Although many antibodies against S protein, especially the RBD have been established, however, the emergence of variants of the SARS-CoV-2 virus has enhanced immune escape and made it more transmissible, which has reduced the efficacy of some clinical neutralizing monoclonal antibodies (mAbs) [6,7,8,9].

Hames et al. [10] reported their first discovery of the naturally occurring heavy-chain-only antibodies (HcAbs) which were devoid of light chains in the camel serum. Based on genetic engineering techniques, variable domains of the heavy-chain of heavy-chain antibody (VHH) were screened and achieved only one domain of the smallest antigen-binding fragment, known as nanobody (Nb) [11]. Nb is the smallest functional antibody fragment ever known to selectively bind to antigens and only one-tenth the size of a conventional IgG antibody. Compared with conventional antibodies, Nb shows some significant advantages, such as the possibility of formatting or multimerization, high affinity, high stability, high solubility, fast tissue penetration, recognition of hidden epitopes, and low production cost, which are regarded as a promising tool for disease diagnosis and treatment, and have the potential to be developed as new diagnostic test reagents for viruses and antiviral drugs [12,13,14]. Potentially useful new analytics for rapid and sensitive SARS-CoV-2 detection have emerged for the diagnosis of COVID-19 serological tests. For example, Guo et al. [15] developed a nanobody-functionalized organic electrochemical transistor (OECT) for rapid quantitative detection of SARS-CoV-2 spike protein in saliva or serum. Laroche et al. [16] applied deep mutation engineering to Nbs, which greatly improved the affinity and extensive cross-reactivity of Nbs to SARS-CoV and SARS-CoV-2 antigens, and cross-neutralized SARS-CoV-2 variants, effectively neutralizing the virus. Previous studies have shown that Nbs monomers or their polyvalent forms were equally or even better than monoclonal antibodies in neutralizing SARS-CoV-2 [17]. The researchers designed Nb21 into trimer form to further enhance its antiviral activity. The Nb was further developed as an inhalable aerosol, named Pittsburgh inhalable nanobody 21 (PiN-21), which is the most effective Nb-neutralizing SARS-CoV-2 [12]. Nanobodies atomized by inhalation can directly reach the viral infection site in the airway, providing faster and stronger antiviral activity and opening up a new drug delivery route for antibody immunotherapy [14,18].

In this study, we constructed a high-quality and specificity phage display VHH library against SARS-CoV-2 RBD, and preliminarily screened four SARS-CoV-2 S-RBD Nbs with different gene sequences. The four Nbs identified by the enzyme-linked immunosorbent assay (ELISA) had excellent specificity and affinity. The results of this study can facilitate the clinical detection, diagnosis, and treatment of SARS-CoV-2 as well as related downstream research.

## 2. Materials and Methods

### 2.1. Reagents and Materials

The SARS-CoV-2 S-RBD protein was purchased from Novamab (Shanghai, China). The SARS-CoV RBD protein was purchased from Biorbyt (Cambridge, UK). The MERS-CoV RBD protein was purchased from prospecbio (Ness Ziona, Israel). The SARS-CoV-2 N protein was purchased from Fapon Biotech (Dongguan, China). Freund’s incomplete adjuvant, horseradish peroxidase (HRP)-conjugated anti-HA monoclonal antibodies (mAbs), anti-mouse IgG-alkaline phosphatase (ALP), Bis (p-nitrophenyl) phosphate (BNPP), and Ni-NTA superflow sepharose column were purchased from Sigma-Aldrich (St. Louis, MO, USA). Mouse anti-HA tag antibody was obtained from Abcam (Cambridge, UK). The *E. coli* TG1 and WK6 competent cells were kept in our laboratory. Anti-SARS-CoV-2 S-RBD mAb (No.bs-41407R) was purchased from Bioss (Beijing, China). 96-well plates were purchased from Merck Corning (Darmstadt, Germany). The SARS-CoV-2 S protein RBD (Omicron), ampicillin (Amp), tetramethylbenzidine (TMB), and isopropyl β-D-thiogalactopyranoside (IPTG) were from Solarbio Biotechnology (Beijing, China).

### 2.2. Camel Immunization

**Figure 1:**
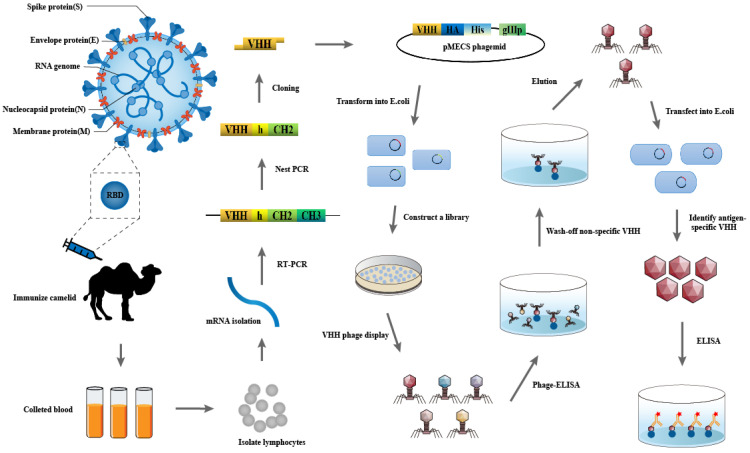
Schematic of the strategy for constructing the immunized library.

The SARS-CoV-2 S-RBD protein was emulsified with an equal volume of Freund’s adjuvant and then immunized with Bactrian camel once every five days for five times to stimulate lymphoid B cells to express antigen-specific nanobodies. Peripheral blood was taken from the camel 3 days after the last immunization, the serum titers were measured and libraries were constructed. The entire schedule is illustrated in Figure 1.

### 2.3. Library Construction

After the last injection, 100 mL of camel peripheral blood lymphocytes were extracted and total RNA was isolated. The extracted RNA was reverse transcribed into cDNA, and then the VHH fragment was amplified by nested PCR. The pMECS phagemid vector and the second PCR product fragment were digested with *Pst*I and *Not*I restriction enzymes and then established a ligation reaction system to ligate the digested vector and the target fragment. The ligation product was electro-transformed into TG1-competent cells to construct a VHH library. TG1 cells repress the amber stop codon between VHH and the gene III in phagemid pMECS, hence VHH-gene III fusion expression. The library size was measured by counting the number of grown monoclonal colonies by gradient dilution. Twenty-four colonies were randomly selected from the library to estimate the correct insertion rate of VHH genes by PCR amplification.

### 2.4. Phage Display for Nanobody Selection

The SARS-CoV-2 S-RBD antigen was selected for library screening based on the results of the post-immunization titration assay. The antigen protein (100 μg/mL) in 100 mmol/mL (mM) NaHCO3 (pH 8.2) was coated in microplates overnight at 4 ℃, while an equal amount of Fc protein was coated as a negative control. The next day, blocking buffer was added to the microplate and incubated at room temperature for 2 h, and antibody crystalline fragment (Fc) protein was added to the amplification library for antigen non-specific antibody adsorption in the same. After washing with PBS with 0.05% Tween-20 (PBST) ten times, the liquid phase adsorbed library was incubated at room temperature for 1 h, and washed 5 times with PBST to remove the non-specific phage. The phage specifically bound to the antigen protein was eluted with triethylamine. The eluted phages were used to infect logarithmic growth *E. coli* TG1 cells. Then they were cultured at 37 °C for 1 h. The process represented one round of bio-panning and the cultured phage was expanded for the next round of screening, and the same screening process was repeated for multiple rounds until the enrichment was obtained.

### 2.5. Periplasmic Extract ELISA (PE-ELISA)

To identify positive colonies, 400 individual colonies from the library were selected randomly for PE-ELISA verification and cultured in terrific broth (TB) medium containing Amp in 24-well plates. After growing to the logarithmic phase, IPTG at a final concentration of 1 mM was added and incubated overnight at 28 °C. The supernatant was collected after an osmotic shock and added to the plate wells which were coated with 5 μg/mL SARS-CoV-2 S-RBD protein overnight in advance, followed by incubation with mouse anti-HA tag antibody for 1 h after washing the unbound antibody with PBST, and subsequently incubated with anti-mouse IgG-ALP after washing with PBST. The chromogenic solution containing bisphosphate (BNPP) was added after washing with PBST. The absorbance was read by a microplate reader (Infinite M200Pro, Tecan, Mennedorf, Switzerland) at 405 nm. The positive colonies were identified when the ratios were higher than 3 and sent to Shanghai Sunny Biotechnology Co., Ltd. (Shanghai, China) for sequencing.

### 2.6. Expression and Purification of Nbs

After sequencing the screened colonies, SARS-CoV-2 S-RBD-specific colonies were amplified and the phagemids of these candidate colonies were extracted and transformed into *E. coli* WK6 cells. WK6 is a non-suppressor strain and expresses the Nb contains a HA-His-tag only. These cells were incubated on Luria-Bertani (LB) medium plates containing Amp overnight at 37 °C. The monoclonals in the plate were selected and inoculated in LB liquid medium and then transferred the strain to amplify it. When the absorbance value at 600 nm is about 0.6–1, IPTG at the final concentration of 1 mM was added and incubated overnight at 28 °C. Bacteria were crushed ultrasonic in the ice bath, the supernatant was purified using Ni-NTA affinity columns using 2 mM imidazole for washing and 20 mM imidazole for elution, and the purity of eluted proteins was analyzed by sodium dodecyl sulfate-polyacrylamide gel electrophoresis (SDS-PAGE) and Western-blot.

### 2.7. Affinity and Specificity Assays

The purified Nbs series diluted by PBS were incubated with 1 µg/mL SARS-CoV-2 S-RBD or RBD Omicron mutant coated on the 96-well enzyme-labeled plates for 1 h at 37 °C. Next, the plates were incubated with the HRP-conjugated anti-HA mouse mAb. The absorbance at 450 nm was read by the microplate reader. The purified Nbs were then added to microtiter plates that were coated with 2 µg/mL SARS-CoV S-RBD, 2 µg/mL MERS-CoV RBD, 2 µg/mL FAP, or 2% BSA and incubated for 1 h at 37 °C. After washing with PBST five times, the bound Nbs were detected by adding 50 μL of HRP conjugate anti-HA mouse mAb. The absorbance at 450 nm was read by the microplate reader.

## 3. Results

### 3.1. Construction of the VHH Library for SARS-CoV-2 S-RBD

**Figure 2:**
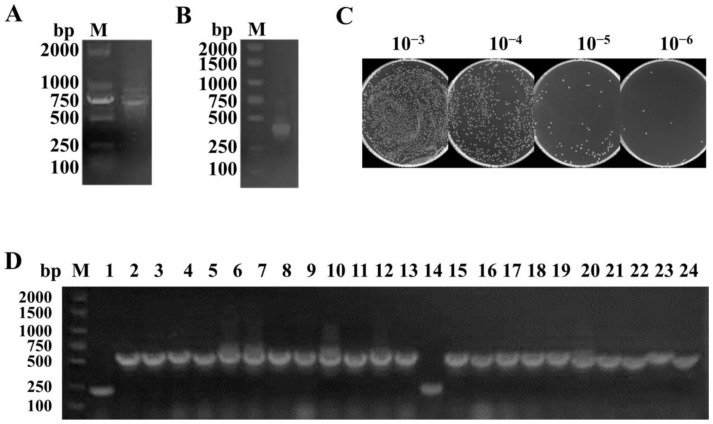
Construction of VHH library. (**A**,**B**) VHH genes were generated by a two-step nested PCR. (**C**) The library size was measured by counting the clone numbers using the gradient dilution method. (**D**) The correct insertion rate was estimated by randomly selecting 24 colonies and performing PCR. M: DNA marker DL 2000.

The first step PCR of the nested PCR obtained a 700 bp DNA band of the VHH-h-CH2-CH3 fragment (Figure 2A), while the second step PCR using the amplified products as DNA templates obtained from the first step obtained another 400 bp band of the VHH exons (Figure 2B). To construct the library, *Pst*I and *Not*I sites were introduced at the 5’ and 3’ ends of the VHH fragments, respectively. In total, VHH fragments and linearized pMECS vector were used for the ligation. Then, the recombinant plasmids were transformed into TG1 cells. The TG1 cells were coated on the plate by gradient dilution, and the library size was determined by counting the number of colonies. The size of the immune library was measured at 4.7 × 10^8^ CFU (Figure 2C). Twenty-four individual colonies were selected randomly for PCR analysis, and the results showed a library insertion rate of 91.7% (Figure 2D), suggesting that a high-quality phage display library for anti-RBD VHHs was obtained.

### 3.2. Screening of the VHH Library for SARS-CoV-2 S-RBD

**Figure 3:**
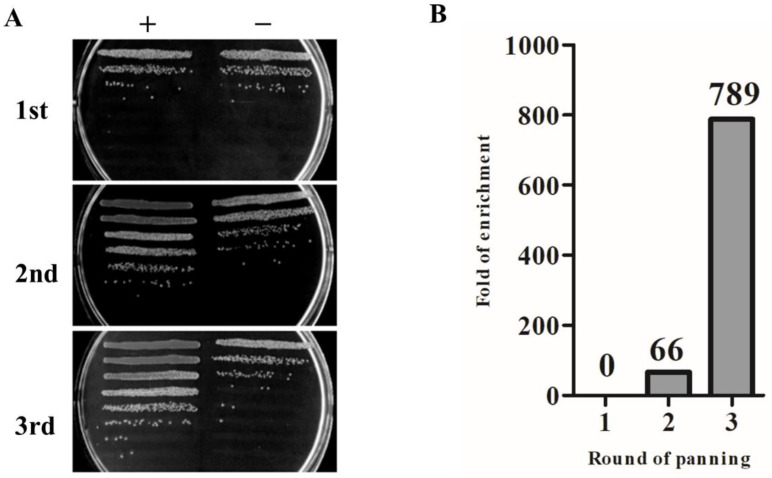
The enrichment effect of SARS-CoV-2 S-RBD phage display nanobody library after affinity screening. (**A**) The enrichment result was displayed for three rounds of panning. (**B**) The statistical result for enrichment fold. +: positive screened phage; −: negative screened phage.

SARS-CoV-2 S-RBD-specific Nbs were identified by bio-panning from the phage display library. In each round, 10 μg of Fc protein was added to 100 μL of phage for non-specific adsorption binding. To evaluate the screening effect, positive screened phage (phage specifically bound to antigen) and negative screened phage (phage bound to Fc) were infected with *E. coli* TG1 in a logarithmic growth phase, and then the bacterial solution was cultured in serial gradient dilutions, and the library enrichment fold (+/−) was calculated by the ratio of the number of colonies. After three rounds of panning, the VHH library was gradually enriched (Figure 3A); the library finally appeared 789-fold enrichment compared with the negative control (Figure 3B).

### 3.3. PE-ELISA Identification of Positive Colonies

**Figure 4:**
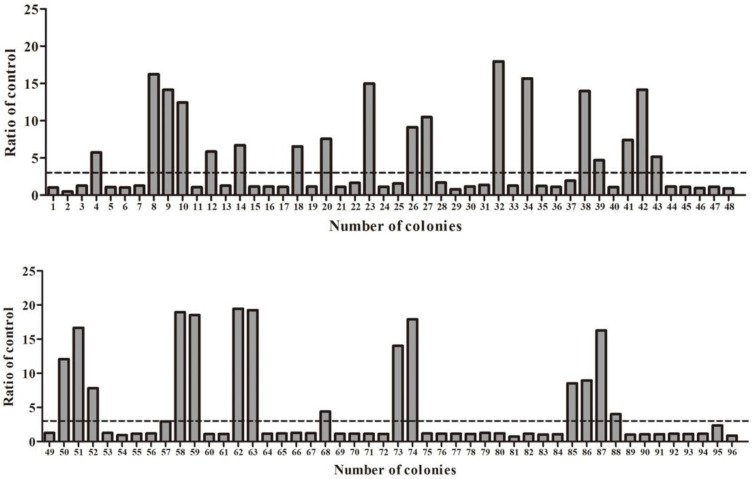
PE-ELISA for the identification of positive colonies. A ratio of higher than 3 was considered positive.

Four hundred colonies were randomly selected and inoculated in a TB medium containing Amp. After the growth of *E. coli* to the logarithmic phase, IPTG at a final concentration of 1 mM was added and incubated overnight at 28 °C. The supernatant was collected after an osmotic shock and transferred to the microplate that had coated antigen overnight in advance, and the coating solution was used as a negative control. The positive colonies were identified when the ratios were higher than 3 and sent to sequencing. The results showed a total of 223 positive colonies, with the ratio (ratio: +/−) ranging from 3 to 20, and some of the identification results (96 of 400) are shown in Figure 4. After PE-ELISA, the sequences of positive colonies were analyzed and then divided into four families (Nb25, Nb52, Nb61, and Nb68) according to the variety of amino acid sequences in CDR3.

### 3.4. Expression and Purification of the Nbs

**Figure 5:**
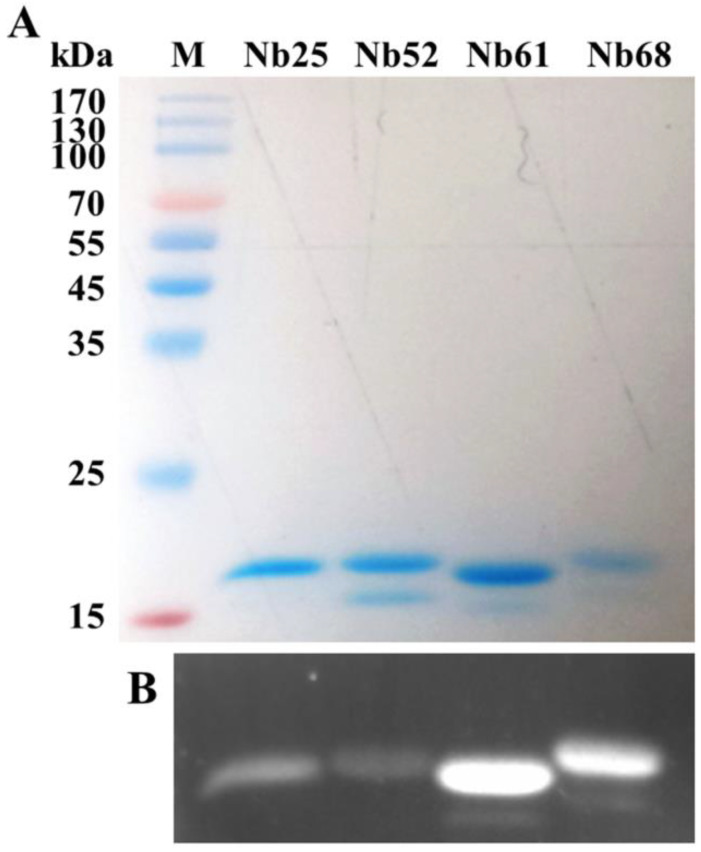
Identification and purification of SARS-CoV-2 S-RBD Nbs. (**A**) SDS-PAGE gel for SARS-CoV-2 S-RBD Nbs (Nb25, Nb52, Nb61, and Nb68). (**B**) Western-blotting assay for confirming the carrying of His-tag of these four Nbs. M: protein marker (10–170 kDa).

Different VHH fragments in the phage display vector pMECS were transformed into *E. coli* WK6 cells. Upon cultured and expanded, soluble Nbs were expressed in the periplasmic region of cells. The induced Nbs were further purified by Ni-NTA superflow sepharose columns. SDS-PAGE analysis showed that Nbs had single bands with high purity (Figure 5A). The molecular weights of the four Nbs were: 14.68 kDa (Nb25), 15.33 kDa (Nb52), 14.75 kDa (Nb61), and 15.11 kDa (Nb68), respectively. The expression of the target proteins was also evaluated by Western blot analysis using Alexa Fluor 680-conjugate anti-mouse mAb and mouse anti-His mAb. The results showed that four Nbs had a specific binding with the tag antibody (Figure 5B).

### 3.5. ELISA to Identify the Affinity and Specificity of Nbs

**Figure 6:**
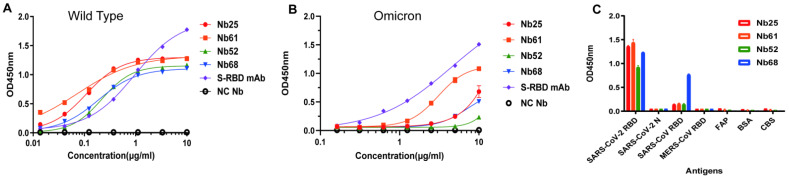
Indirect ELISA to identify the affinity of Nbs to SARS-CoV-2 S-RBD. (**A**) The binding affinity of Nbs to SARS-CoV-2 S-RBD (Wild Type); (**B**) the binding affinity of Nbs to SARS-CoV-2 S-RBD (Omicron); (**C**) the specificity of Nbs to SARS-CoV-2 S-RBD; commercial anti-SARS-CoV-2 RBD mAb was used as the positive control. The OD450 values were the means ± SD values from triplicate measurements.

The affinity of the four recombinant proteins to SARS-CoV-2 S-RBD was detected by ELISA. All of the selected Nbs showed a high absorbance value at 450 nm, and the EC50 of Nbs was lower then 0.25 µg/mL, demonstrating that the selected Nbs had a high affinity to SARS-CoV-2 S-RBD (Figure 6A). Among the four Nbs strains, Nb61 had the highest binding affinity to SARS-CoV-2 S-RBD whose EC50 <0.1 µg/mL. However, the Nbs screened also face the risk of reduced binding affinity for SARS-CoV-2 Omicron variants. All four strains of Nbs showed varying degrees of decreased binding for Omicron (Figure 6B). The specificities of the four Nbs were determined via ELISA by comparison with cross-reactions with other proteins, including SARS-CoV-2 N, SARS-CoV S-RBD, MERS-CoV S-RBD, FAP, and BSA. The results showed a specific binding activity with SARS-CoV-2 S-RBD with no other cross-activity detected in the ELISA results except for Nb68 (Figure 6C).

## 4. Discussion

SARS-CoV-2 poses a serious threat to global public health due to its high infectiveness, rapid transmission, being widespread, and having an alarming mutation rate. Previous studies have shown that early convalescent plasma (CP) treatment was able to prevent clinical deterioration, but it was limited by the supply of plasma from survivors. Neutralizing mAbs can reduce the risk of death in patients with COVID-19, however, it is not widely available because of their high cost, as they must be made in tissue and cell cultures [19]. The exploration of Nb with a broad neutralizing effect provides a new direction for the development of potential drug candidates for COVID-19 [18,20,21].

In this study, we constructed a VHH library targeting SARS-CoV-2 S protein and screened four Nbs by using routine phage display technology; it was preliminarily verified the high binding specificity and affinity of four Nbs to SARS-CoV-2 S-RBD protein (Wild Type and Omicron) by Western-blot and ELISA. Among them, Nb61 had the highest affinity to the SARS-CoV-2 S-RBD protein (Wild Type) (EC50 < 0.1 μg/mL). Although the binding ability of the selected Nbs to the Omicron variant of SARS-CoV-2 was slightly decreased, it was not completely lost. Linking the Nbs to a heterogeneous Nbs may improve their affinity [15]. Our results provide a valuable basis for the development of rapid and sensitive immunodiagnostic methods and therapeutic drugs for SARS-CoV-2. 

Like conventional mAbs, the VHH genes can be cloned into eukaryotic or prokaryotic expression vectors, and proteins of VHHs can also be prepared by a variety of prokaryotic and eukaryotic expression systems, with advantages of lower production cost but higher yield than mAbs [9,10,11]. Due to its characteristics of small molecular weight, high affinity, and ability to bind a wide range of epitopes, Nb has higher detection sensitivity than conventional antibodies and is considered to be a substitute for mAb in rapid pathogen detection and disease diagnosis [22]. Since the passive diffusion rate of molecules in tissues is approximately inversely proportional to the molecular size, Nb has deep and fast tissue penetration compared with conventional antibodies and is easy to be directly delivered to the tissues of infected patients’ respiratory systems utilizing aerosol inhalation [14,19,23]. Previous studies have shown that the construction of Nb in multivalent form can increase its stability, prolong the half-life in vivo, and improve its neutralization activity with the SARS-CoV-2 to suppress mutational escape [24]. Li et al. [15] found that a bispecific antibody bn03 with a broad binding capacity of SARS-CoV-2 mutants could be effectively delivered to the lungs via inhalation administration, significantly reducing virus titers in the lungs of mice infected with SARS-CoV-2 and improving the pathological state. The efficacy of Nb against SARS-CoV-2 infection has been demonstrated in cell and animal studies [25,26]. In summary, with the continuous progress of antibody-drug screening and production technology, Nb has developed into a highly versatile molecule with extensive biological and clinical application value due to its superior molecular characteristics. As a potential means of disease diagnosis and treatment, Nb plays an increasingly important role in the prevention and treatment of infectious diseases. The unique technical advantages of Nbs will play a broader role in more areas [24,27,28,29].

However, some limitations should be considered in this study. The binding of the Nbs to additional SARS-CoV-2 variants outside of Omicron was not tested, and the epitope diversity and function of the Nbs obtained need to be further explored. In order to screen Nbs with better performance, we still need more panning and screening experiments, such as virus neutralization tests. The result of SDS-PAGE showed Nb 52 and Nb61 had multiple bands that may be due to contamination of the samples or protein degradation caused by prolonged sample storage or repeated freeze-thawing. It is important to continue to establish and perfect multiple prokaryotic and eukaryotic expression and purification systems of Nbs, to ensure the correct modification and functional activity of proteins, and to improve the solubility, purity, and yield of Nbs prepared in vitro. In addition, since Nbs can be easily fused and modified by genetic engineering technology, we will develop Nb complexes with SARS-CoV-2 S-RBD-specific Nbs as the core of the technology. We have already developed and prepared the Nb61-enzyme complex and Nb61-reporter gene complex, which can be used for various SARS-CoV-2 detection methods in the future. We will also develop Nb-multimer complex or Nb-targeted drugs, and conduct animal studies in the future to test the protective effect of Nbs in animals.

## Data Availability

The data that support the findings of this study are available from the corresponding author upon reasonable request.
